# Prevalence of *Plasmodium* Species Using Rapid Diagnostic Tests in Cameroon: First‐Year Data From Routine Malaria Surveillance

**DOI:** 10.1155/bmri/6605623

**Published:** 2026-04-15

**Authors:** Raymond Ngomdjum Tabue, Flora Neh, Elvira Evina, Dominique Amougou Bomba, Junior Voundi Voundi, Jean Fosso, Albert Meka Zeh

**Affiliations:** ^1^ Prevention Section, Cameroon National Malaria Control Program, Yaoundé, Cameroon; ^2^ Cameroon National Malaria Control Program, Ministry of Public Health, Yaoundé, Cameroon, minsante.gov.cm; ^3^ Monitoring and Evaluation Section, Cameroon National Malaria Control Program, Yaoundé, Cameroon; ^4^ Case Management Section, Cameroon National Malaria Control Program, Yaoundé, Cameroon

**Keywords:** Cameroon, malaria, *Plasmodium*

## Abstract

**Background:**

Malaria remains a major public health concern in Cameroon. Although *Plasmodium falciparum* is the predominant parasite, the prevalence of other *Plasmodium* species remains poorly documented due to the absence of a formal surveillance system. This study is aimed at assessing the prevalence of *Plasmodium* species through routine malaria surveillance using P.f/Pan rapid diagnostic tests (RDTs).

**Methods:**

A nationwide malaria surveillance program was conducted over 1 year (January–December 2024), covering health facilities across all regions and levels of care. Malaria cases were diagnosed using P.f/Pan RDTs, which detect *P. falciparum* and non‐*falciparum* infections but cannot reliably differentiate individual non‐*falciparum* species. Data were systematically compiled through the National Health Information System and analysed to determine the prevalence and distribution of *Plasmodium* species across regions.

**Results:**

Of the 3,843,507 individuals tested, 43% were malaria‐positive. *P. falciparum* monoinfection accounted for 82% of cases, mixed infections (*P. falciparum* + Pan) for 15%, and Pan infection for 3%. Regional analysis showed the highest prevalence of *P. falciparum* monoinfection in the Far North region (92.1%), followed by the North (85.7%), Central (82.5%), West (81.7%), and East (80.6%) regions. Seasonal variations revealed a peak in positive cases between June and July, whereas mixed and Pan infections remained consistently low.

**Conclusions:**

Findings confirm the presence of non‐*falciparum* and mixed infections beyond *P. falciparum.* However, P.f/Pan RDTs have inherent limitations in species differentiation, underscoring the need for molecular techniques to improve species‐specific identification. Strengthening malaria genomic surveillance, including monitoring of *Plasmodium* species diversity and HRP2/3 gene deletions that affect RDT performance, will be critical to enhance routine surveillance and guide effective malaria control strategies in Cameroon.

## 1. Introduction

Globally, malaria is a major cause of morbidity and mortality, with the sub‐Saharan Africa region bearing most of the burden [1]. In 2023, 263 million malaria cases and 597,000 malaria deaths worldwide were reported. Approximately 95% of the deaths occurred in sub‐Saharan African countries, where many people at risk still lack access to the services they need to prevent, detect, and treat the disease [2]. Malaria accounted for 29% of outpatient visits and 40% of deaths among children under five in Cameroon in 2023 [3]. The national parasite prevalence is 26.1%, with significant regional disparities, particularly in forested and rural areas [4]. Overall, transmission is mesoendemic, although in some areas it is still hyperendemic (mainly in forest areas in the southern part of the country) [5]. With an estimated population of 29,442,327 inhabitants in 2024 [6], the entire population of Cameroon is at risk of the disease. Cameroon′s 10 regions, grouped into four geoecological zones (Sudano Sahelian, savanna, coastal, and tropical forest), show a rainfall decreasing from the south to the north, shaping malaria transmission patterns [7–10].

The national strategy for malaria control prioritises prevention and case management [11, 12]. To ensure quality care, health facilities are regularly supplied with diagnostic tools and antimalarial drugs, complemented by continuous training of health personnel. Amongst the five *Plasmodium* species involved in malaria transmission [2, 13], four have been described in Cameroon (*Plasmodium vivax*, *P. malariae*, *P. ovale*, *and P. falciparum*), with *P. falciparum* accounting for approximately 95% of infections [14–18]. Transmission is sustained by at least 17 *Anopheles* species [8, 19]. Studies also suggest that malaria diagnosis relies heavily on rapid diagnostic test (RDT), which detects only *P. falciparum* [11, 12]. This limitation, compounded by reagent shortages and poor microscopy quality, has hindered species‐specific surveillance.

Across Africa, the emergence of drug‐resistant malaria strains, particularly resistance to ACTs, is a growing concern. These resistant strains, already widespread in the Greater Mekong Subregion, have already begun to emerge in Eastern Africa, threatening to reverse decades of progress in malaria control [20, 21]. Since the 2000s, ACTs have been at the heart of Cameroon′s malaria control strategy, significantly reducing the disease burden. However, this heavy reliance has also increased selection pressure on the malaria parasite, raising concerns about the sustainability of current treatment approaches. To address this challenge, strengthening parasite surveillance has become a priority. Since 2023, the country has opted for an update of the Monthly Activity Report (MAR) of health facilities through a disaggregation of data relating to *Plasmodium* species resulting from diagnosis by RDTs and microscopy. This study presents data from routine surveillance 1 year after this update.

## 2. Materials and Methods

### 2.1. Organization of the Health System

Cameroon′s health system follows a three‐phase pyramidal structure comprising central, intermediate, and peripheral levels. At the central level are the Ministry of Public Health and central and general hospitals. The intermediate level consists of 10 regional delegations that provide technical support to health districts. The peripheral level includes 203 district health services responsible for implementing health programs. Health facilities are classified into six categories, ranging from Category 6 (health facilities) to Category 1 (national referral hospitals).

### 2.2. Study Population

The study population included all outpatients presenting to health facilities across the country between January and December 2024 who were tested for suspected malaria using the P.f/Pan RDT. Individuals were included if they had a recorded RDT result in consultation registers and subsequently reported in the MAR. Exclusion criteria were incomplete records, missing RDT results, or cases not entered into the District Health Information System 2 (DHIS2).

### 2.3. Study Design and Data Extraction

**Figure 1:**
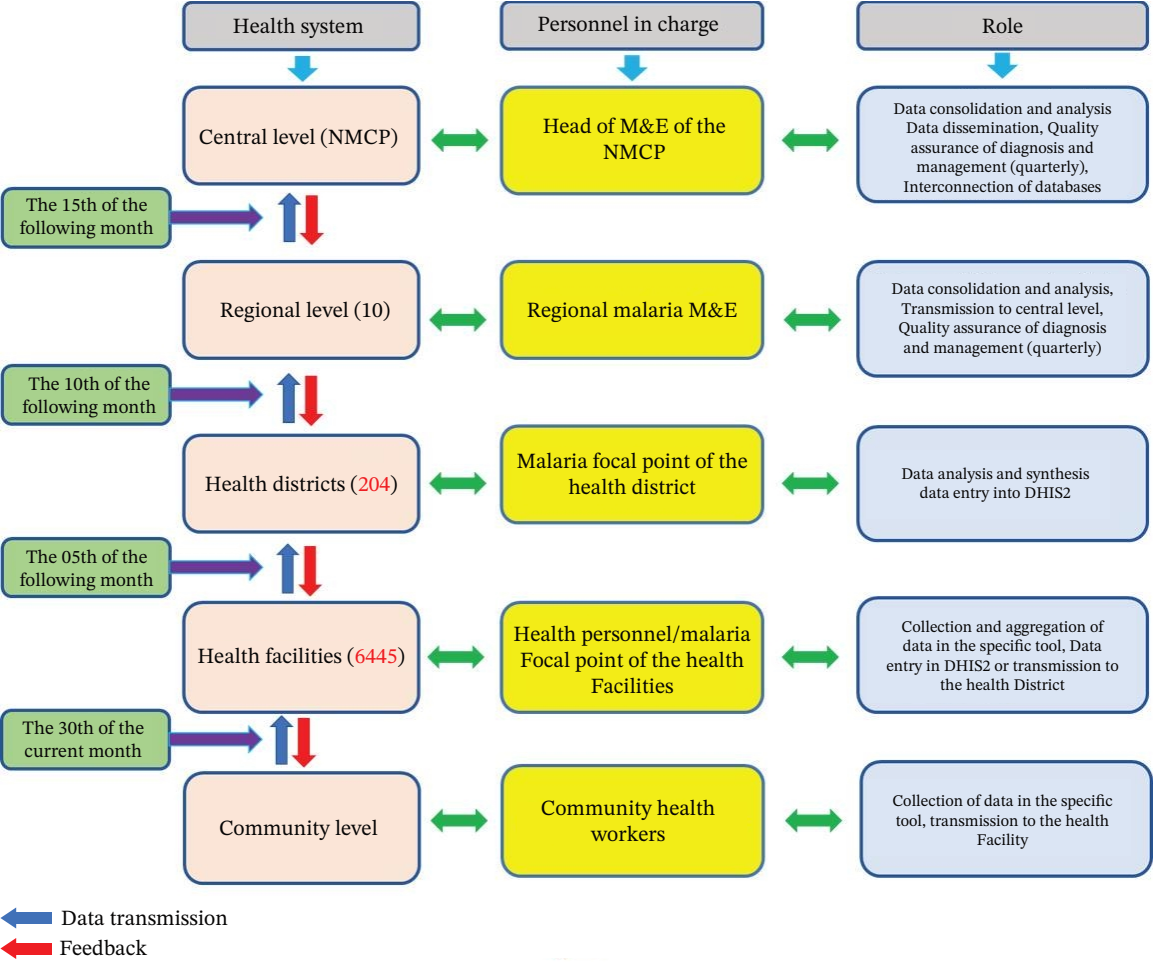
Routine malaria surveillance data flow.

A retrospective analysis of routine surveillance data was conducted. Malaria RDT results from consultation registers were aggregated monthly in MAR and entered into DHIS2 (Figure 1). In 2023, the MAR was updated to disaggregate malaria cases by *Plasmodium* species: (i) *P. falciparum* only, (ii) mixed infections (*P. falciparum* + Pan), and (iii) non‐*falciparum* species (Pan). The national database for 2024 was downloaded from DHIS2 on 21 February 2025 and transferred into Excel for analysis.

### 2.4. Malaria Case Detection and *Plasmodium* Species Identification

Passive case detection was performed in health facilities using the Bioline Malaria Ag P.f/Pan test from ABBOTT. This RDT detects histidine‐rich protein 2 (HRP2) antigen specific to *P.f* and Pan‐*Plasmodium* lactate dehydrogenase (pLDH) antigens. A patient was considered malaria positive if the RDT showed: (i) *P. falciparum* only, (ii) *P. falciparum* + Pan, or (iii) Pan only. Although this test allows differentiation between *P. falciparum* and non‐*falciparum* species, it does not identify individual non‐*falciparum* species.

### 2.5. Quality Control and Data Management

Quality assurance measures included adherence to manufacturer guidelines for RDT use, periodic supervision of health facilities, and cross‐checking MAR entries against consultation registers. Data consistency was verified during DHIS2 extraction, and duplicates and incomplete entries were excluded.

### 2.6. Statistical Analyses

Data were analyzed using descriptive statistics to summarise malaria cases by category, region, and month. Temporal trends were assessed using the chi‐square tests for proportions and linear regression to assess incidence trends. Statistical significance was set at *p* < 0.05.

## 3. Results

### 3.1. Health Map Analysis

Cameroon′s health map comprises 10 regions with 203 health districts, 1999 health areas, and 6454 health facilities (Table 1). These health facilities are grouped into six categories, of which integrated health facilities account for 85.9% (5542/6454), followed by 9.41% (607/6454) of district medical centers, and 4.17% (269/6454) for the district hospital. Regional, central, and general Hospitals together accounted for only 0.56% (36/6454).

**Table 1:** Organization of the health system in Cameroon in 2024.

Region	Health district	Health area	Health facility						
			Category 1: General hospital	Category 2: Central hospital	Category 3: Regional hospital	Category 4: District hospital	Category 5: District medical centers	Category 6: Integrated health facility	Total
			*n*(%)	*n*(%)	*n*(%)	*n*(%)	*n*(%)	*n*(%)	*n*
Adamawa	11	109	0 (0%)	1 (0.4%)	1 (0.4%)	13 (5.6%)	23 (10.0%)	193 (83.5%)	231
Centre	32	333	2 (0.1%)	5 (0.2%)	1 (0.0%)	52 (2.6%)	206 (10.2%)	1749 (86.8%)	2,015
East	15	176	0 (0%)	1 (0.3%)	2 (0.7%)	19 (6.4%)	33 (11.0%)	244 (81.6%)	299
Far North	32	341	0 (0%)	0 (0%)	4 (0.8%)	30 (6.3%)	31 (6.6%)	408 (86.3%)	473
Littoral	24	186	2 (0.2%)	1 (0.1%)	2 (0.2%)	41 (3.8%)	97 (8.9%)	949 (86.9%)	1,092
North	15	146	0 (0%)	1 (0.3%)	2 (0.6%)	15 (4.5%)	17 (5.1%)	301 (89.6%)	336
Northwest	21	242	0 (0%)	0 (0%)	2 (0.5%)	24 (5.9%)	46 (11.2%)	337 (82.4%)	409
West	20	239	0 (0%)	1 (0.1%)	2 (0.2%)	34 (3.5%)	91 (9.4%)	836 (86.7%)	964
South	12	110	0 (0%)	2 (0.6%)	1 (0.3%)	13 (4.1%)	35 (11.1%)	264 (83.8%)	315
Southwest	21	117	0 (0%)	0 (0%)	3 (0.9%)	28 (8.8%)	28 (8.8%)	261 (81.6%)	320
**Total**	**203**	**1,999**	**4 (0.1%)**	**12 (0.2%)**	**20 (0.3%)**	**269 (4.2%)**	**607 (9.4%)**	**5,542 (85.9%)**	**6,454**

### 3.2. Completeness of Clinical Consultation and Diagnostic Variables

**Figure 2:**
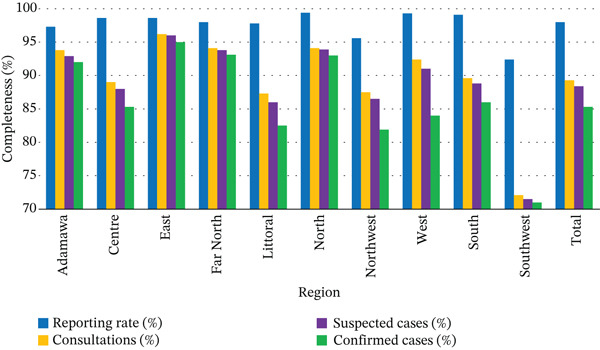
Completeness of clinical consultation and diagnostic variables.

Overall, reporting completeness was high, reaching an average of 98% nationwide (Figure 2). The North (99.4%) and West (99.3%) regions show the best results. Completeness varied across consultations, suspected cases, and confirmed cases, with regional disparities observed. Indeed, some regions, such as East, Far North, and North, record high levels of completeness (> 93% for consultations, suspected cases, and confirmed cases). Conversely, the southwest region had the lowest completeness across all variables (around 71%). The difference between consultations, suspected cases, and confirmed cases shows a slight progressive decline in the completeness of each category, with the national average ranging from 89.3% for consultations to 85.3% for confirmed cases (Figure 2).

### 3.3. Malaria Epidemiological Data and *Plasmodium* Species Prevalence

In 2024, a total of 9,534,279 people consulted in the country′s health facilities, 41% (3,926,248/9,534,279) were suspected malaria cases. Among the 3,926,248 malaria suspected cases, 98% (3,843,507/3,926,248) were tested either by RDT or by microscopy (Table 2). Analysis of positive malaria tests showed that *P. falciparum* accounted for 97% (1,608,624/1,664,988) of infections, comprising 82% (1,364,057/1,664,988) monoinfections and 15% (244,567/1,664,988) mixed infections with *P. falciparum +* Pan species. Other *Plasmodium* species (Pan positive) contributed 3% (56,364/1,664,988) of infections. Regional analysis of *Plasmodium* species prevalence shows a consistent pattern, with the highest prevalence of *P. falciparum* monoinfection observed in the Far North region (92.1%; 298,736/324,479), followed by the North (85.7%; 150,044/174,997), Centre (82.5%; 310,561/376,602), West (81.7%; 117,646/143,951), and East (80.6%; 102,883/127,721) regions.

**Table 2:** Summary of malaria RDT‐positive tests across Cameroon in 2024.

Region	No. of health facility consultation	No. of malaria‐suspected cases	No. of malaria‐suspected cases tested	Malaria positive test by RDT			
				Total	RDT *P.f* positive	RDT *P.f* + Pan positive	RDT Pan positive
				*n*	*n*(%)	*n*(%)	*n*(%)
Adamawa	446,835	235,516	229,957	97,962	70,596 (72%)	22,538 (23%)	4,828 (5%)
Centre	2,101,788	785,999	76,296	376,602	310,561 (82%)	53,437 (14%)	12,604 (3%)
East	479,978	242,082	232,803	127,721	102,883 (81%)	20,488 (16%)	4,350 (3%)
Far North	1,418,169	751,315	741,104	324,479	298,736 (92%)	22,155 (7%)	3,588 (1%)
Littoral	1,810,192	595,819	579,113	183,976	145,614 (79%)	31,786 (17%)	6,576 (4%)
North	640,656	400,339	394,469	174,997	150,044 (86%)	22,447 (13%)	2,506 (1%)
Northwest	874,721	250,912	247,901	92,267	61,694 (67%)	22,841 (25%)	7,732 (8%)
West	764,721	317,379	314,228	143,951	117,646 (82%)	21,881 (15%)	4,424 (3%)
South	288,912	120,224	118,310	58,185	44,991 (77%)	11,076 (19%)	2,118 (4%)
Southwest	708,307	226,663	220,326	84,848	61,292 (72%)	15,918 (19%)	7,638 (9%)
**Total**	**9,534,279**	3,926,248	**3,843,507**	**1,664,988**	**1,364,057 (82%)**	**244,567 (15%)**	**56,364 (3%)**

**Figure 3:**
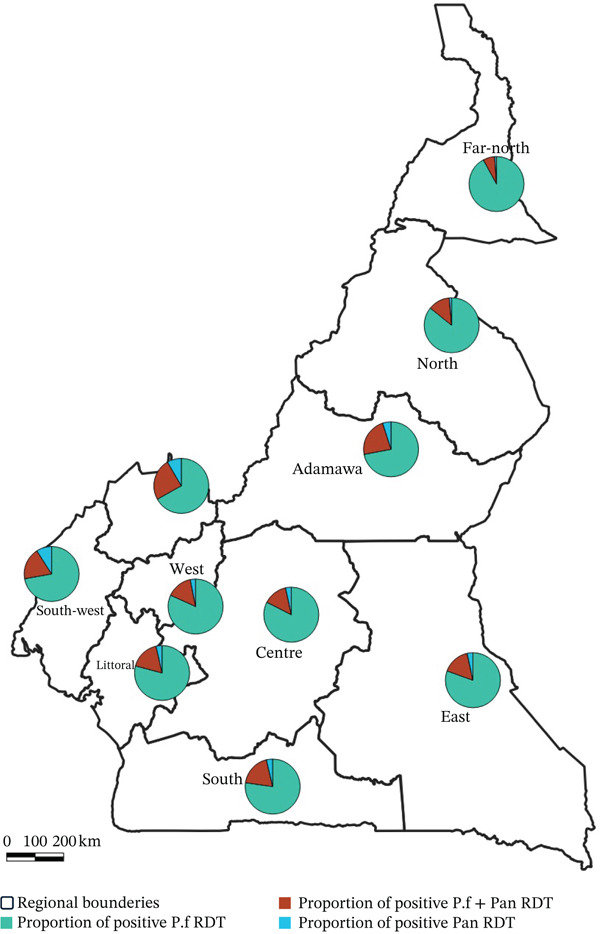
Geospatial distribution of *Plasmodium* species prevalence across the country in 2024.

The prevalence of mixed infection (*P. falciparum* + Pan) was higher in the Northwest (24.8%; 22,841/92,267), followed by Adamawa (23%; 22,538/97,962), Southwest (19%; 15,918/84,848), and South (18.8%; 11,076/58,185). In contrast, Pan‐positive infections were more frequent in the Southwest (9%; 7638/84,848), Northwest (8.4%; 7732/92,267), and Adamawa (4.9%; 4828/97,962) (Figure 3). Statistical comparison of the mean number of *P. falciparum*–positive RDTs, *P. falciparum* + Pan–positive RDTs, and Pan‐positive RDTs revealed significant differences among groups (*p* < 0.05).

The analysis shows significant correlations observed among categories, *P. falciparum*–positive RDTs versus *P. falciparum* + Pan–positive RDTs (*r* = 0.710) and *P. falciparum* + Pan–positive RDTs versus Pan‐only RDTs (*r* = 0.794).

### 3.4. Temporal Analysis of the Prevalence of *Plasmodium* Species

**Figure 4:**
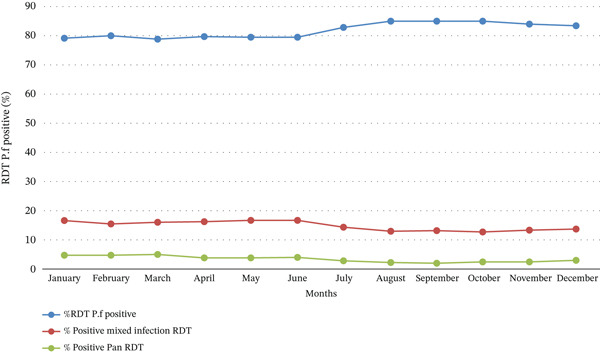
Monthly variation of *Plasmodium* species in 2024.

A descriptive temporal trend shows a significant increase in the percentage of *P. falciparum*–positive RDTs between June and July, peaking at 84% before stabilizing (Figure 4). Unlike *P. falciparum* alone, the percentage of mixed infections (*P. falciparum* + Pan) remained relatively low and stable, suggesting that *P. falciparum* remains the dominant species. The percentage of Pan‐positive RDTs gradually decreased from January to June before rising again towards the end of the year.

### 3.5. Analysis of *Plasmodium* Species Prevalence by Health Facility Category

The analysis of *Plasmodium* species prevalence by health facility category reveals that 80.9% (1,347,165/1,664,988) of malaria‐positive tested cases by RDT originated from integrated health facilities, 11.38% (189,409/1,664,988) from district medical centers, 6.78% (112,822/1,664,988) from district hospitals, and 0.94% (15,592/1,664,988) from other categories (Table 3).

**Table 3:** Summary of malaria RDT positivity by health facility category in Cameroon in 2024.

Health facility category	No. of RDT *P. falciparum* positive (%)	No. of RDT *P.f* + Pan positive (%)	No. of RDT Pan positive (%)	Total *n*(%)
Category 1: General hospital	287 (0.02)	30 (0.002)	11 (0.001)	328 (0.02)
Category 2: Central hospital	759 (0.05)	81 (0.005)	149 (0.009)	989 (0.06)
Category 3: Regional hospital	9,932 (0.60)	2,421 (0.15)	1,922 (0.12)	14,275 (0.86)
Category 4: District hospital	89,150 (5.35)	17,216 (1.03)	6,456 (0.39)	112,822 (6.78)
Category 5: District medical centers	148,716 (8.93)	31,474 (1.89)	9,219 (0.55)	189,409 (11.38)
Category 6: Integrated health facility	1,115,213 (66.95)	193,345 (11.61)	38,607 (2.32)	1,347,165 (80.91)
Total	1,364,057 (81.9)	244,567 (14.7)	56,364 (3.4)	1,664,988 (100)

## 4. Discussion

The present study is aimed at evaluating the national prevalence of *Plasmodium* species by analyzing the P.f/Pan RDT results carried out on patients in the country′s health facilities in 2024. *P. falciparum* monoinfection accounted for 82%; mixed infection (*P*. *falciparum* + Pan) represented 15%; and Pan‐only infections were detected in 3%. Although *P. falciparum* was the predominant species causing malaria in the Cameroonian population, other species were still frequently present among patients with malaria symptoms. The analysis shows significant correlations between *P. falciparum*–positive RDTs and *P. falciparum* + Pan–positive RDTs, and between *P. falciparum* + Pan–positive RDTs and Pan‐only RDTs. These correlations show close statistical associations but should be interpreted cautiously, as they do not imply causality; rather, they highlight co‐occurrence patterns useful for surveillance and intervention planning.

The Southwest region shows the lowest completeness for clinical consultation and diagnostic variables, likely due to ongoing disruptions in the healthcare system stemming from political instability since 2018. A slight decrease in completeness was observed across consultations (89.3%), suspected cases (88.4%), and confirmed cases (85.3%), which may be due to common challenges faced by healthcare facilities in reporting outpatient and laboratory data. Variation in reporting completeness could bias species prevalence and infection trend estimates. In particular, the Southwest region, where completeness was lowest, likely underrepresented malaria cases due to health system disruptions.

To effectively monitor malaria in the country, a sentinel surveillance system was established with the creation of 35 sentinel sites. This sentinel surveillance targets malaria vectors and parasites. Although vector surveillance is implemented in these sites, parasite surveillance is absent. This parasite surveillance should consider *Plasmodium* species and parasite resistance to antimalarial drugs. In the absence of a surveillance system for *Plasmodium* species, cross‐sectional studies conducted in the country reveal the presence of *P. falciparum*, *P. malariae*, and *P. vivax* [16–18]. Across studies, *P. falciparum* consistently predominates among species malaria infections, with a proportion sometimes reaching 95% [14, 15, 18]. This corroborates the results obtained in the present study, where the prevalence of *P. falciparum* monoinfections varied from 92.1% in the Far North to 67% in the North West. In addition, *P. falciparum* was involved in 15% of coinfections.

Other *Plasmodium* species accounted for 18% overall, that is, 15% in coinfection with *P. falciparum* and 3% other *Plasmodium* species. However, the reliance on P.f/Pan RDTs may contribute to an underestimation of non‐*falciparum* species. Although these RDTs are designed to detect *P. falciparum* and pan‐malarial antigens, their sensitivity for species such as *P. malariae*, *P. ovale*, and *P. vivax* is lower compared with microscopy or PCR. Consequently, the predominance of *P. falciparum* observed in routine data may partly reflect diagnostic limitations. In fact, previous studies show that the prevalence of *P. malariae* is the highest in the country after *P. falciparum*, up to 4% in monoinfection [18, 22, 23]. In East Africa, some studies associated the declining *P. falciparum* prevalence with increasing non‐*P. falciparum* infections [24–26]. Coinfections with *P. falciparum + P. malariae* are frequently encountered with prevalences ranging from 5.96% to 13.95% [18, 22, 23]. Regarding *P. vivax,* many studies pointed out its presence in Cameroon [16, 17, 27]. In the West region, *P. vivax* infection was detected by PCR in 5.6% (27/484) patients [16]. According to data from another study in the Southwest region, 14.9% (13/87) of *Plasmodium* infection cases were exclusively or concomitantly due to *P. vivax* [17]. Furthermore, in the literature, 4% of malaria infections were due to *P. vivax* in mono and mixed infections according to a study conducted in Bertoua in the East region, Yaounde in the Centre region, Kye‐ossi and Ebolowa in the South region, and Douala in the Littoral region [28].

Although detected in the country, the distribution of *Plasmodium* species in general and that of *P. vivax* in the country is unclear. The current routine data do not allow for disaggregation by *Plasmodium* species. However, the circulation of *P. vivax* in the country has implications for malaria treatment. Indeed, unlike other malaria parasite species, the treatment of patients infected with *P. vivax* requires therapy at both the blood stages of the parasite (blood schizontocidal) and prevention of subsequent relapses (hypnozoitocidal), as the parasite can form dormant liver stages (hypnozoites) capable of causing relapsing infections weeks to months after the initial blood stage infection [29].

In the absence of *P. vivax* diagnostic tool in health facilities, all the malaria‐positive patients are treated the same with ACTs, without taking into account the *Plasmodium* species. Furthermore, by considering *P. falciparum* as the only malaria parasite circulating in the country, some funders do not finance the acquisition of malaria species diagnostic tools.

Cameroon lacks genomic surveillance of malaria parasites, which limits the identification of emerging resistance patterns and genetic changes affecting diagnostic performance. HRP2/3 gene deletions, increasingly reported in Africa, can compromise the sensitivity of RDTs specific to *P. falciparum*, leading to false negatives and underestimation of the malaria burden. The deficiencies in genomic surveillance prevent detection of these deletions, together with other species‐specific markers, which results in decreased parasite surveillance capacity and species identification capabilities.

During the last decade, the proportion of *P. falciparum* and pan‐malaria RDTs meeting the current WHO performance threshold for procurement increased from approximately 20% to 90% [30]. Furthermore, the latest‐generation RDTs used for *P*. *vivax* diagnosis have sensitivities comparable with microscopy [30]. By considering the prevalence of *P. vivax* in some studies in some regions, approximately 1%–4% of *P. vivax*–infected patients do not receive appropriate malaria treatment. This may partly explain malaria treatment failures. Cameroon′s current policy regarding malaria case management is multiple first‐line treatments (MFT). This MFT consists of two or more first‐line ACTs deployed simultaneously. This approach mitigates the emergence and spreading of resistance by making it harder for parasites to develop resistance [31]. However, the current MFT strategy is primarily designed to target *P. falciparum* infections. It does not explicitly account for non‐*falciparum* species or mixed infections, which remain present across the country. In the absence of species‐specific diagnostic and treatment protocols, patients infected with *P. malariae*, *P. ovale*, *or P. vivax* are managed with ACTs intended for *P. falciparum*, potentially limiting treatment effectiveness and contributing to therapeutic failures.

## 5. Study Limitation

The main limitation of the study lies in the lack of a breakdown of the prevalence of each of the *Plasmodium* species. This is due to the availability of only P.f/Pan RDTs. Furthermore, only patients tested by RDT were considered. This situation is explained by the diagnostic policy implemented in the country, which recommends that microscopy be performed only in Categories 1–5 health facilities. However, these health facilities represent only 19.1% of all health facilities in the country. As for Category 6 health facilities, where diagnosis is systematically performed by RDT, they represent 89.9%. These results highlight the predominance of Integrated Health Facilities in the dataset, underscoring the need to interpret percentages cautiously and in relation to facility type. Stratification by facility category is essential to avoid misinterpretation and to ensure that trends observed in smaller facility types are not obscured by the disproportionate contribution of Integrated Health Facilities. In addition, due to reagent shortages, electricity supply disturbances, and skilled personnel, Category 1–5 health facilities also perform RDTs in addition to microscopy.

## 6. Conclusions

Malaria remains a significant public health challenge in Cameroon, and first‐year surveillance data provide crucial insights into the distribution of *Plasmodium* species across the country. Although *P*. *falciparum* is the dominant species, the findings confirm the presence of non‐*falciparum* species, emphasizing the complexity of malaria transmission in the country. This study underscores the limitations of current diagnostic techniques, specifically P.f/Pan RDTs, which may fail to accurately differentiate among various *Plasmodium* species. Consequently, there is a growing need for molecular techniques to enhance species‐specific identification, ensuring more accurate diagnosis and effective treatment strategies. Malaria genomic surveillance in Cameroon faces critical gaps requiring urgent attention. These gaps include challenges in monitoring the prevalence of *Plasmodium* species, tracking HRP2/3 gene deletions that affect the reliability of RDT performance, assessing the extent of antimalarial drug resistance, and understanding vector resistance to insecticides. Addressing these gaps through improved surveillance is essential to strengthen malaria control. By incorporating genomic surveillance strategies, researchers and policymakers can systematically monitor malaria trends, allowing for informed decision‐making based on scientific evidence.

**Nomenclature**
RDTs: Rapid diagnostic tests for malaria testing offer a rapid and accurate method for diagnosing malaria by detecting specific antigens produced by malaria parasites in the blood. RDTs are particularly useful in settings where good‐quality microscopy services are not readily available. They are available in various formats and are based on the lateral flow or immunochromatographic strip method, which detects the presence of malaria antigens by a color change on a nitrocellulose strip.
ACT: Artemisinin‐based combination therapy is the recommended treatment for malaria caused by *Plasmodium falciparum*. ACTs consist of two or more drugs with different modes of action, including artemisinin derivatives (dihydroartemisinin, artesunate, and artemether) combined with other drugs (lumefantrine, mefloquine, amodiaquine, sulfadoxine/pyrimethamine, piperaquine, and chlorproguanil/dapsone).
MAR: The monthly activity report is a booklet that contains all the health system variables of interest on priority illnesses, including malaria. This booklet is filled in by data managers in health facilities on a monthly basis. All the variables are therefore entered into the National Health Information System through the District Health Information Systems 2 (DHIS2) application. The data entered are therefore accessible at the higher levels of the health pyramid. To ensure the data quality, the data entered were regularly reviewed and validated at the health district level (monthly) and the regional level (quarterly). Feedbacks were regularly provided to health facilities to correct any inconsistencies.
ITN: A net (usually a bed net) that has been treated with a safe residual insecticide designed to physically block mosquitoes, which carry the malaria parasite. The residual insecticide is designed to last for up to 3 years.

## Author Contributions

R.N.T. conceived the study and was involved in the design, coordination, analysis, and drafted the manuscript. F.N. and E.E. were involved in the drafting and reviewing of the manuscript. D.A.B. and J.V.V. reviewed the article. J.F. and A.M.Z. were involved in organizing and analyzing the data, interpreting the findings, and reviewing the article.

## Funding

No funding was received for this manuscript.

## Disclosure

All authors read and approved the manuscript.

## Consent

All the authors approved the final version of the manuscript for publication.

## Conflicts of Interest

The authors declare no conflicts of interest.

## Data Availability

The data that support the findings of this study are available from the corresponding author upon reasonable request.
